# Carcinoma of unknown primary (CUP) of the head and neck: state of the art and future perspectives

**DOI:** 10.3389/fonc.2026.1813394

**Published:** 2026-05-20

**Authors:** Sara Farinatti, Alberto Paderno, Caterina Giannitto, Cristina Gurizzan, Silvia Uccella, Luigi Lorini, Maria Ausilia Teriaca, Fabio Ferreli, Marcello Rodari, Carlo Resteghini, Armando Di Bello, Giuseppe Mercante, Giuseppe Spriano, Paolo Bossi

**Affiliations:** 1 Medical Oncology and Hematology Unit, IRCCS Humanitas Research Hospital, Milan, Italy; 2 Department of Biomedical Sciences, Humanitas University, Milan, Italy; 3 Otorhinolaryngology Head & Neck Surgery Unit, IRCCS Humanitas Research Hospital, Milan, Italy; 4 Radiology Division, IRCCS Humanitas Research Hospital, Milan, Italy; 5 Pathology Service IRCCS Humanitas Research Hospital, Milan, Italy; 6 Department of Radiotherapy and Radiosurgery, IRCCS Humanitas Research Hospital, Milan, Italy; 7 Department of Nuclear Medicine, IRCCS Humanitas Research Hospital, Milan, Italy; 8 Medical Oncology, Università Cattolica del Sacro Cuore, Comprehensive Cancer Center, Fondazione Policlinico Gemelli, IRCCS, Rome, Italy

**Keywords:** carcinoma of unknown primary, head and neck cancer, personalization, squamous cell carcinomas, trans oral robotic surgery

## Abstract

Despite advances in molecular oncology, head and neck cancer diagnosis still relies on anatomical site and histopathology. In up to 5% of metastatic cases, no identifiable primary tumor is found, defining carcinoma of unknown primary (CUP) and posing significant diagnostic and therapeutic challenges. We conducted a narrative review of the literature focusing on current diagnostic and treatment strategies for head and neck CUP, including imaging, viral biomarkers (HPV, EBV), minimally invasive procedures, and transoral robotic surgery (TORS). While TORS plays a dual role in diagnosis and treatment, a multidisciplinary approach of neck dissection, radiotherapy, and chemotherapy in high-risk patients, remains the cornerstone of management. Personalization is increasingly crucial in this comprehensive context, mainly involving treatment tailoring and radiotherapy planning, where limited evidence on field definition is available. Ongoing trials, such as UK FIND study, highlight the potential of targeted surgery to tailor and possibly reduce radiotherapy fields for better patient outcomes and improved quality of life.

## Introduction

While a personalized and molecular-based era of oncology is developing, we must come to terms with the idea that the cornerstone of oncological diagnosis still relies on two major factors: the anatomic area of origin and the morphological characteristics of cancerous cells. Appropriate treatment choices require a primary location and histological confirmation.

In rare cases, cancer cells are found in the absence of clear primary lesions. The detection of a secondary localization in the absence of a clear primary cancer configures an entity known as Carcinoma of Unknown Primary (CUP), which is generally characterized by a poor prognosis (1).

In terms of numbers, the phenomenon accounts for around 1-2% of all cancer diagnoses, decreasing from 3-5% in historical data (2), owing to advances in diagnostic strategies.

Despite this encouraging data, some anatomic areas appear to be more affected than others. In the head and neck region, for example, CUPs can represent up to 5% of all diagnoses (3).

In terms of histology, adenocarcinomas are the most represented subset of all CUPs, accounting for around 50% of all cases, while squamous cell origin is found in only 15%. Nevertheless, when focusing on the head and neck region, squamous cell carcinomas represent up to two-thirds of all CUP cases (4).

Considering these data, two primary considerations arise: on the one hand, defining the histotype is not enough, as treatment choices and efficacy data are still strongly linked to the site of origin; on the other hand, whenever evidence of a primary is lacking, a tendency to intensify therapeutic interventions and treatment choices arises, increasing the risk for over-treatment. In the head and neck region, longing for better local control may carry an especially high burden of acute and long-term toxicities due to collateral damage to structures fundamental in several daily physiological functions.

De-intensification strategies in head and neck squamous cell carcinomas (HNSCC) are already under investigation when a primary lesion is evident, with the aim to reduce toxicities while maintaining efficacy and overall survival results (5). This is especially true for human papillomavirus (HPV)-related cases, which have an intrinsic better prognosis if compared with HPV-nonrelated counterparts (6). A high need for personalization and integration of treatment strategies in this particular branch of oncology is therefore mandatory.

The absence of an accurate disease picture in HNSCC CUPs configures a slippery slope, where a difficult balance between surgery, radiation volume, doses and fields, and use of systemic therapy is often required, and a high risk for overtreatment stands right around the corner.

## Aiming for primary’s diagnosis

Absence of a clear primary transcribes into a difficulty in establishing a therapeutic approach and a consequent overall strategy. The first step in overcoming the issue resides in an extensive quest for the primary lesion, as retrospective evidence suggest the presence of a benefit in both disease-free survival (DFS) and overall-survival (OS), when a primary lesion is identified (7). In this context, imaging is of fundamental importance, although not always successful. Conventional radiology with CT scans, although representing the first level imaging, may not be enough, as small and superficial mucosal lesions can be hard to detect, and MRI may be more sensitive in identifying the primary lesion; however, the second level radiological approache 18-FDG-PET-CT scan is often indispensable to achieve a conclusive diagnosis (8).

### Diagnostic work-up

According to a recent systematic review, the optimal strategy involves utilization of different imaging modalities: starting from a contrast-enhanced CT and MRI guided management, which can detect up to 44% of occult tumors, moving on to 18-FDG-PET-CT, which represents the most sensitive imaging modality for the identification of CUPs. These radiological exams should therefore be considered prior to panendoscopy (9).

Imaging interpretation requires careful evaluation of the shape, margins, and enhancement pattern of the lingual and palatine tonsils; on MRI the primary lesion may only be identified on Diffusion Weighted Imaging (DWI), given background tissue suppression that may increase the conspicuity of cellular primary tumor (10).

When compared with other imaging modalities, 18-FDG-PET-CT carries a high sensitivity (73.1%) and negative predictive value (68.9%) for primary site detection in CUPs, it is prone to false positives and false negatives and has limited overall accuracy, especially for small primary tumors (11). False-positive results on 18-FDG PET are higher after tonsillar manipulation, mainly when performed after a prior intervention (12); according to evidence from small perspective studies, such imaging should therefore preferably be performed before tissue sampling, also potentially enabling for PET-directed bioptic sampling (13, 14).

Trends in the diagnostic imaging of CUPs in the head and neck area are evolving; a recent HNCIG and IFHNOS survey by Williamson et al., encompassing responses from 48 global experts, revealed a high consensus on the use of outpatient laryngoscopy (81.3%), contrast-enhanced CT (77.1%), and 18-FDG-PET-CT (79.2%) as frontline imaging modalities in the diagnostic workup of CUPs; MRI on the other hand was routinely used by only 50% of respondents, indicating variability in its perceived utility, possibly due to accessibility, interpretation challenges, or redundancy with PET-CT findings. These trends underscore a preference for imaging techniques that combine anatomical detail with functional insights, particularly PET-CT, which plays a pivotal role in detecting metabolically active occult disease (15).

**Figure 1:**
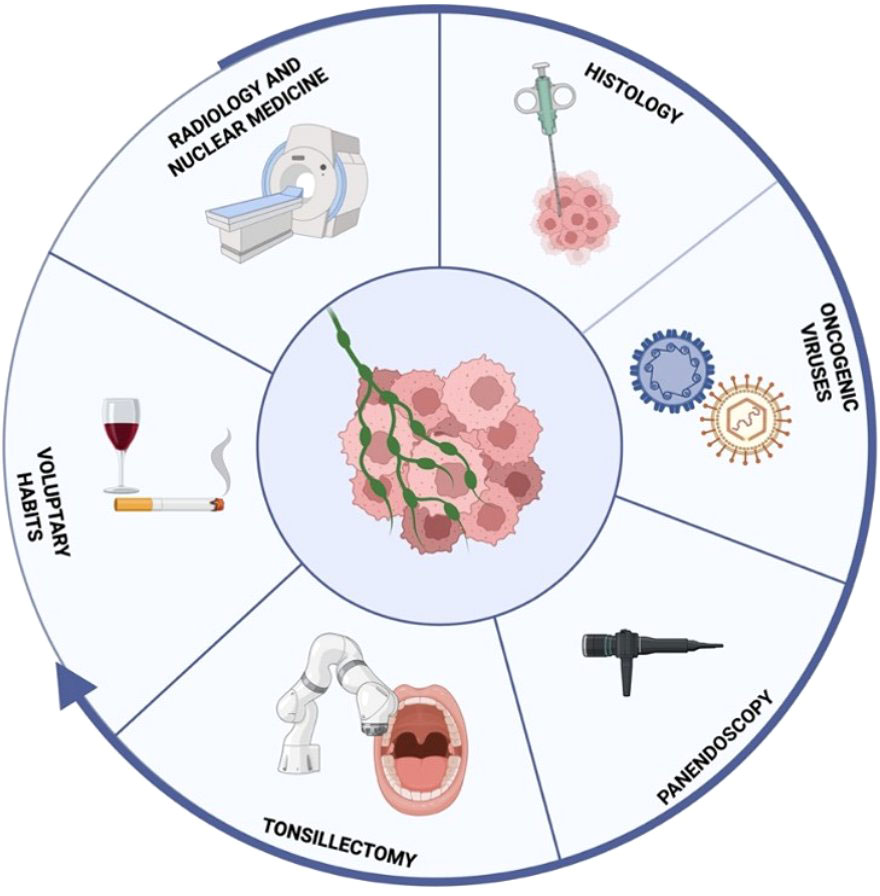
Diagnostic work-up of HNCUPs starts from the association of conventional and nuclear imaging to the histological diagnosis of a squamous cell carcinoma. Subsequent efforts need to focus on finding potential viral etiological agents, pointing toward an anatomical district of origin, followed by endoscopic and, eventually, surgical diagnostic procedures; specifically, Trans Oral Robotic Surgery can bear both a diagnostic and a therapeutic value. Along the whole process patients’ characteristics maintain a strong importance in guiding treatment and diagnostic choices.

Whenever imaging is unsatisfactory, interventional approaches can be implemented as surrogates:

- Following a complete radiologic staging, Fine Needle AgoBiopsy (FNAB) aided by ultrasound guidance, is mandatory. This procedure allows for a quick and safe collection of a sample amenable for histopathologic analysis, representing the cornerstone for a correct diagnosis, as opposed to either cytological studies or nodal exeresis (16, 17).Strong evidence concerning the best technique to obtain an histo-pathological diagnosis is lacking; with current guidelines endorsing the use of FNAB as the gold standard, as it yields a diagnostic performance comparable to both core biopsies (18) and node exeresis in the diagnosis of SCCs, while still allowing to perform immunohistochemical analysis on the sample, and maintaining a relative low risk for procedure-related extra nodal disease seeding as well as procedure-related complications (19). Surgical node exeresis, once used as the standard approach, has progressively been abandoned, being a more invasive procedure, and carrying a higher rate of periprocedural complications, mainly bleeding and secondary infections; with some suggesting for it to be associated to a higher risk of surgical field disruption, with a consequent negative impact on subsequent therapeutic approaches and an increase in the rate of both local and distant recurrences (20, 21). This contrasts with lymphoproliferative disorders, intrinsically different diseases when compared to solid tumors, where the gold standard is still represented by surgical node exeresis, as the analysis of the whole pathological lymph nodes, of their architecture, their topography and of the sub-clonal composition of the lymphoid infiltrate, can increase diagnostic accuracy (22).

- In the molecular characterization-era, where histological categories are increasingly dissected and integrated by genomic analyses, some other features may be of use to define primary tumor location, even in absence of radiological evidence. Dealing with squamous cell histology in the head and neck region warrants for the search of oncogenic viruses in both the histological specimen and blood samples, owing to both a prognostic and, potentially, a predictive value. In absence of a certain primary, finding a viral etiology may raise the clinical suspicion for a specific area of origin, allowing for a disease diagnosis:
  - I. Human papillomavirus (HPV) is commonly associated with oropharyngeal squamous cell carcinoma (OPSCC) development (23). When a clear primary cannot be identified with conventional radiological methods, HPV presence allows to define an anatomical origin for cancer occurrence: in its VIII edition, published in 2017, the American Joint Committee on Cancer (AJCC)’s Tumor Node Metastasis (TNM) classification, therefore classified p16 positive CUPs as oropharyngeal in origin (24).
  - Immunohistochemically detected p16 overexpression in the histological sample is generally used as a surrogate marker for a biologically active HPV infection in OPSCC cancers, allowing to infer HPV causation (24). Regardless, the use of p16 in CUPs does not seem to be an appropriate predictor for SCC primary location, as its positivity has been reported also in around 20% of lymph node metastasis from skin SCC (25) and non-SCC histotypes such as small cell neuroendocrine carcinomas (NECs) (26), suggesting an integration with HPV DNA assessment through polymerase chain reaction (PCR) is still required in this specific entity (27). If on one hand HPV positivity represents an etiological factor, its detection also marks a positive prognostic indicator in the context of head and neck SCC (28). Recently, in a small series of patients, the accuracy of circulating tumoral HPVDNA (ctHPVDNA) was studied in comparison to standard diagnostic procedures to define the HPV status, authors were able to conclude that ctHPVDNA has a good reliability in identifying an HPV-positive primary cancer (29).
  - III. Epstein-Barr virus (EBV) is known to be associated to 95% nasopharyngeal carcinoma cases in endemic areas and around 20% in non-endemic areas (30). While HPV CUPs are reported to account for up to 60% of all cases, EBV positivity is reported to be much rarer: in a prospective study published in 2003, out of 12 SCC CUPs cases EBER positivity through *in situ* hybridization (ISH) was detected in only one case (31). Current guidelines therefore suggest testing for EBV positivity only in HPV negative cases, with the VIII edition of AJCC’s TNM classification considering EBV positive CUPs as nasopharyngeal in origin (28).
- Panendoscopy of the upper aerodigestive tract is a diagnostic procedure performed under general anesthesia; it includes a combined rhinoscopy, a nasopharyngoscopy, the inspection of the oral cavity and oropharynx, a direct laryngoscopy, hypopharyngoscopy, esophagoscopy, and, lastly, a bronchoscopy. The aim is to visualize the whole mucosa, in search of potentially malignant areas to biopsy, bearing also in mind the concept of ‘field cancerization’ as a known carcinogenic process in development of squamous cell cancer of both the head and neck and esophageal areas. As a tendency for the two entities to occur in a synchronous or metachronous fashion does exist, a full endoscopic evaluation has consequently been proposed as an aid in both the differential diagnosis and the exclusion of a second primary (32). According to 2020 ASCO guidelines a complete upper aerodigestive tract examination (oral cavity, naso-oro-hypopharynx and larynx) is always indicated to visualize the mucosa and consequently allow for a biopsy of suspicious areas (33, 34). Multiple random biopsies, on the other hand, are not indicated as they do not improve diagnostic accuracy of the procedure (33). Regardless, the yield of extensive endoscopic studies in localizing a primary in CUPs is not clearly established, with retrospective evidence suggesting a low diagnostic accuracy (around 20-30%) (11, 12); in a coherent way no current indication is present for such a comprehensive diagnostic workup in the context of head and neck SCC (35).
- Any endoscopic procedure, even solely laryngoscopy, can be aided by the use of Narrow Band Imaging (NBI), a promising technique allowing for image-enhanced endoscopy and a consequent better visualization of the mucosal vascularization and of areas suggestive for malignancy. In a recent meta-analysis in the context of head and neck CUPs, this diagnostic strategy is reported to improve detection rate by around 35% when applied to various endoscopic approaches (36). This may aid in the detection of small oropharyngeal primary tumors, a common source of false-negative results on 18-FDG-PET-CT due to the obscuration by physiologic up-take in the normal lingual or palatine tonsillar lymphoid tissue.
- In a stepwise approach, whenever endoscopic evaluations turn out negative, as the evidence of affected SCC nodes may underlie an oropharyngeal primary, **tonsillectomy** must be considered (37). Detection rates increase when palatine tonsillectomy is coupled to lingual tonsillectomy, moving from 32% up to 64% (38). In particular, transoral robotic surgery (TORS) has been proven to be an effective strategy to improve detection in the oropharyngeal area (39), as well as overall survival in a large HPV positive retrospective series of HNCUPs (40). According to 2020 ASCO guidelines, ipsilateral tonsillectomy can be considered whenever panendoscopy, coupled to directed biopsies, fails to show a primary, as contralateral tonsillectomy carries a higher risk for complications and sequelae, such as edema, increased risk of bleeding, and postoperative pain, while being associated with a low probability of unveiling the primary tumor. While SCC of the tonsil tends to metastasize to the cervical region, disease spread to contralateral lymph nodes in absence of ipsilateral nodal disease is in fact exceptional (41). As synchronous bilateral tonsil carcinomas are limited to case reports, current evidence does not support the routine use of this bilateral approach, reserving it to highly selected cases and following a multidisciplinary discussion. For what concerns tongue base mucosectomy (TMB), it may be reserved for patients with no histologically confirmed disease following tonsillectomy or for patients with bilateral lymphadenopathies, owing to the higher probability of an oropharyngeal origin (36). A recently published small prospective trial on the subject suggested a significant improvement in detection rate following TMB, reaching 50% of the enrolled cases, and being highest when performed in patients who had already undergone an historic tonsillectomy (42).
  Both palatine and lingual tonsillectomy have a role that goes beyond diagnosis, guiding and being part of the treatment plan. Locating the tumor primary allows for its removal with free margins, thus offering a strong rationale for treatment de-intensification on the specific area, avoiding or reducing radiotherapy dose, with a consequent decrease in potential long term side effects.
- Lastly, often under-looked, whenever radiology and interventional approaches fail to define a clear site of origin, the patient’s characteristics must be balanced out in association with HPV and EBV status in the diagnostic algorithm. **Voluptuary habits**, such as smoking status and heavy alcohol consumption may acquire a vital relevance, pointing more toward oropharyngeal origin as compared to the nasopharynx (**Figure 1**) (43).

## Treatment strategies

Whenever diagnostic efforts are unsuccessful, therapeutic approaches are lent from sub-sites and are related to the specific location of the secondary lesion, in an attempt to avoid a ‘primary emergence’. At present, an effort toward personalization is still required, as strong evidence is lacking and strategies are heterogeneous (44). While no prospective trial exists on the matter, current therapeutic choices are based on retrospective evidence, on the supposed primary location and on the different secondary sites in the head and neck region (45).

The two pillars in managing SCC CUPs, as extrapolated from data obtained in the broader context of HNSCC, reside in surgery and radiotherapy, in either a monomodal fashion or interlinked in a multi-modal strategy. Whilst chemotherapy’s role is still open to discussion and mainly limited to association approaches (35).

### Surgery

As mentioned, the integration of transoral robotic surgery (TORS) in the diagnostic work-up has redefined the diagnostic and therapeutic landscape for HPV-associated CUPs: TORS-guided lingual and palatine tonsillectomy not only allows for identification of the primary site but also enables risk-adapted treatment. A 2024 Danish cohort study of 153 early-stage oropharyngeal cancers treated with primary TORS and neck dissection reported 5-year overall survival of 87.6% and recurrence-free survival of 84.9%, despite 88.9% of patients avoiding adjuvant therapy entirely (46). These outcomes underscore the dual role of TORS in achieving oncologic control while minimizing treatment-related dysphagia (long-term feeding tube rates: 1.8% vs. 20% in salvage cases) (47).

Emerging protocols advocate for TORS as a first-line diagnostic tool in HPV-associated CUPs, since it reduces reliance on empiric radiotherapy. When combined with PET-CT, TORS increases diagnostic sensitivity by 18% with respect to conventional panendoscopy (48). This precision allows surgeons to limit neck dissection extent, as bilateral procedures are reserved for radiologically confirmed contralateral disease (14% of cases), and to tailor adjuvant regimens based on margin status and pathological extranodal extension (ENE) findings (49, 50).

**Figure 2:**
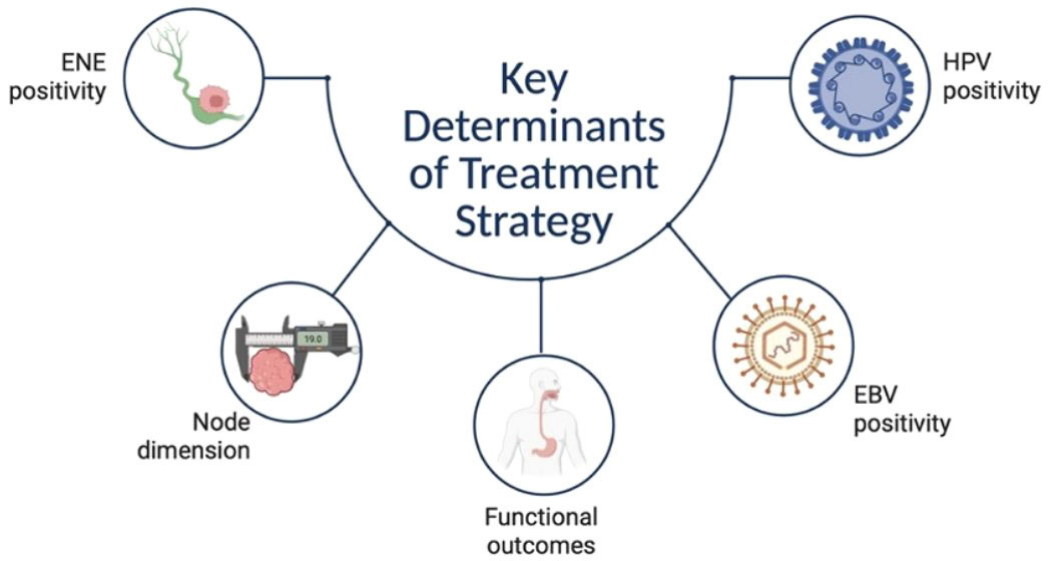
ENE: extra nodal extension. Key decision factors contributing to multidisciplinary tailoring, guiding physicians in the integration of the different treatment options in CUPs, involve both disease extension according to TNM and extra nodal involvement, as well as the presence of etiological viral agents and the long-term functional impact determined by the use of a multimodal approach.

The associated lymph node neck dissection represents the cornerstone of surgical management; while no randomized prospective studies directly comparing selective neck dissection (SND) and modified radical neck dissection (MRND) exist, in clinical practice MRND is often favored in case of advanced nodal burden or radiographic suspicion of extranodal extension (ENE). Surgical intervention is prioritized when radical resection is feasible, particularly for ipsilateral cN1 disease (<3 cm), without radiological evidence of ENE. The objective is to achieve a comprehensive lymphatic clearance while potentially reducing the need for adjuvant therapies (51). For more advanced nodal involvement (cN2/N3), surgery remains a critical component of multimodal treatment, though it necessitates postoperative radiotherapy (PORT), with concurrent chemotherapy limited to high-risk features such as ENE or positive margins (**Figure 2**) (51).

Consequently, the presence of pathologic ENE significantly influences adjuvant therapy decisions, with rates escalating from 17% in cN1 to 26% in cN2b disease. Notably, cN2b cases carry a threefold higher risk of pathological ENE positivity when compared to cN1–cN2a disease (52). At the same time, in the Pathos cohort of 291 HPV-positive OPSCC, radiological ENE positivity was reported to carry a very low sensitivity (at best 56%), with a higher specificity (70% at worst), for pathological ENE detection (53).

In case of HPV-positive disease, patient selection for surgery requires an even more meticulous risk stratification, as tri-modality therapy (neck dissection ± tonsillectomy followed by chemo-radiation) harbors a heightened functional morbidity when compared to bimodal approaches (surgery and PORT or definitive chemo-radiation) (51).

### Radiotherapy

Its role is either upfront, with radical intent, or post-operative, in the adjuvant setting. In the first case scenario the decision to spare an up-front surgical approach, and its consequent side effects, comes from retrospective evidence suggesting that surgery alone, in presence of high burden or high-risk diseases, does not yield sufficient loco-regional results; as a consequence, radiotherapy at curative dose may offer a better local control, leaving an open door for a potential salvage surgery in case of a local recurrence (54). According to the latest ASCO guidelines, definitive chemoradiotherapy is favored in cases where surgery would be extensive, consequently increasing morbidity as well as resort to tri-modality approaches; this is mainly the case of large-volume, bilateral lymph nodes cancers and/or in case of evident extranodal extension (33).

Retrospective studies and large metanalysis on HNSCC oropharyngeal in origin demonstrated a correlation between radiological and pathological ENE positivity and an increase in loco-regional recurrences, as well as in incidence of distant metastasis; when limiting our analysis to HPV-related diseases, both radiological and pathological ENE showed a strong correlation with prognosis. In large retrospective and registry studies, the presence of ENE conferred a worse prognosis, thus justifying a different staging classification (55, 56).

In the post operative, adjuvant, setting the choice to add radiotherapy is therefore considered whenever known risk factors, such as positive margins, as well as pathological ENE, are found on histology (44). Even if some data indicate that in HPV-related OPSCC ENE below 1 mm may be treated with a de-escalated approach of radiation without chemotherapy, more evidence on the topic is needed before this assumption may be adopted in real life (57).

Many other questions remain open in the setting, the most important being radiotherapy fields and the consequent irradiated volumes; current practice stands on bilateral neck irradiation, possibly associated to mucosal irradiation, longing for the inclusion of the unknown primary into the treatment fields, reserving mono-lateral irradiation to strictly lower risk feature, unilateral disease (36, 58). The aim of a unilateral irradiation resides on evidence from small retrospective cohort studies, proving lower incidence of acute side effects, such as xerostomia and dysphagia, when compared to the more extensive bilateral approach, without a significant impact on either disease recurrence or survival; long term follow-up remains limited (58, 59). A large prospective trial, the EORTC-24001-22005, was designed to clearly answer this question, randomizing SCC CUPs to either ipsilateral or extensive radiotherapy, involving a bilateral neck and mucosal irradiation; however, the study was unable to achieve a sufficient patient accrual, leading to its closure and leaving this fundamental question unanswered.

Treatment de-intensification and, conversely, intensification, remains an up to date and understudy concept, currently lacking corroboration.

Validation of predictive and prognostic biomarkers, either pathological or clinical, may guide us in the choice, as retrospective evidence suggests that unselected patients display worst outcomes in terms of both nodal and mucosal relapse, whenever a unilateral approach is favored (58). A cost-benefit discussion should therefore be considered, putting together the extension of radiation fields, the consequent expected toxicities and the biological characteristics we already tackled, as well as the information obtained from operative diagnostic procedures such as tonsillectomy and lingual mucosectomy.

### Chemotherapy

Chemotherapy regimens are confined to the margin of CUPs therapeutic approaches, as their role in association to radiotherapy is limited to regionally advanced disease in a definitive setting. While the trimodal strategy should be avoided in the initial decision making, as it is burdened by higher acute and late side effects, concurrent chemoradiation remains indicated for pathological high-risk cases, such as postoperative evidence of ENE positivity and/or positive margins (35). No current evidence suggests chemotherapy can be omitted or reduced in its dosage in case of HPV-positive nodes, in absence of a corresponding clinical and/or radiological primary, with high dose cisplatin remaining the preferred regimen in association to radiotherapy.

Borrowing treatment strategies from the expected primary sites, an induction chemotherapy course followed by concomitant chemoradiation may be considered in EBV positive, extensive nodal involvement cases, as their nasopharyngeal counterparts (45).

## Balancing outcomes and toxicity burden: de-escalation

As we have seen so far, several histological and biological characteristics of the tumor have proven to be associated to different outcomes. Current treatment strategies in presence of an oropharyngeal primary are trying to focus on de-escalation for HPV positive cases, although neither clinical nor biological predictors of efficacy are currently present and ctDNA analysis, to tailor therapeutic approaches, is still under investigation. While no phase III trials are available yet, results from preliminary studies hold great promise (60). De-escalation approaches may therefore also be applied to HPV positive nodes in absence of an overt primary site, regardless further verification through rigorous clinical trials is mandatory, as for the case of known primary tumors.

### The UK experience: FIND trial

A potential translation of the oropharyngeal de-intensification trend in CUPs’ context does exist and comes from the recent results of the FIND phase II clinical trial (61). Focusing exclusively on HPV positive cases, transoral robotic surgery (TORS) offered a tool for both diagnostic and therapeutic purposes. An interventional diagnostic approach on tumor primary, tailored on HPV positivity, was proven to appropriately aid in diagnosis, allowing for subsequent de-intensification approaches on the secondary nodal area.

The idea is to favor a monomodal approach to the nodal area, either surgery or radiotherapy, where feasible. Intensification in the diagnostic process proved effective for a subsequent de-intensification in the therapeutic intervention; as the detection of a primary lesion during TORS, in the absence of negative prognostic histological features (ENE, nodes > 3cm in size or positive margins), was coupled to pharyngeal-sparing radiotherapy, with a consequent positive long-term impact on swallowing.

This United Kingdom experience imparts two principal insights. Firstly, HPV-associated CUPs, by their very nature, demonstrate an intrinsic potential for enhanced outcomes and benefit from the advocated de-intensification strategy. Conversely, it becomes apparent that, in attempt to generalize this approach to the entire category of SCC-associated CUPs, it is imperative to customize treatment strategies and sequences in accordance with clinical attributes, risk factors, and the proposed site of origin. This trial enrolled a relatively small cohort of patients, with a total of 22 patients analyzed: 13 in the diagnostic-intent arm and 9 in the therapeutic-intent one. Further multicenter phase III studies are therefore warranted to validate these preliminary findings; meanwhile, in absence of strong prospective evidence, a proposed well-balanced approach certainly has to rely on EBV and HPV presence, but subsequent tailoring on radiological and pathological evidence of both disease nodal burden and ENE remains crucial. Configuration of different risk categories allows to define where surgery and radiotherapy can be used alone and when they need to be integrated, bearing in mind that interventional surgical approaches, such as TORS, are already configuring a possible tool in the diagnostic setting, allowing for a therapeutic approach on the hidden primary and sparing aggressive untargeted procedures.

While some of the factors we have reviewed can help us tailor a diagnostic and, hopefully, also a therapeutic approach to the primary tumor, a second intervention will be required on the evident secondary site of nodal involvement. Mono-modal approaches can be considered in the absence of high-risk features; in all other occasions, multi-modality remains the cornerstone, striving toward a reduction in radiotherapy volumes and confining tri-modal approaches for HPV-negative cases.

## Discussion and relevance

To wrap up, few certainties are available in this distinctive head and neck SCC setting; with current efforts focusing on potential biomarkers of origin and on therapeutic de-intensification.

HPV-related disorders, known for their more favorable prognosis and the consequent potential for de-intensification approaches, configure a special category in both the oropharyngeal and CUPs landscapes, and consequently deserve special attention in the multidisciplinary setting.

For what concerns HPV negative patients, in the specific case of CUPs, where worst survival is observed in comparison to their known primary counterparts, no novel strategies are currently available. Especially in presence of high-risk characteristics, a trimodal approach of surgery followed by chemo-radiotherapy must be considered, as this subgroup of patients represents a class where intensification efforts may be worthy (62).

If on one hand HPV will likely represent, in the next future, the cornerstone for head and neck treatment de-intensification, in the tailoring of different therapeutic strategies for SCC CUPs, other factors should also be integrated. Of all things, the role of radiological and pathological ENE on both loco-regional and long-term outcomes must be further explored, especially in the context of HPV positivity (63). In the attempt to de-intensify HPV positive cases, sparing a trimodal approach, radiological evidence of ENE positivity can make us question the need for a surgical cervical neck dissection, as the pathological evidence may prevent us from sparing the post-operative chemo-radiotherapy.

Lastly, when dealing with de-intensification, we must not forget radiotherapy’s fields, doses and volumes: incorporation of histological features, viral loads, as well as of the results of diagnostic procedures, such as tonsillectomy and base of the tongue mucosectomy, is fundamental in order to depict clinical and biological heterogeneity, unveiling complexity and to allowing for adequate treatment tailoring.

It is growingly evident that CUPs configure complex entities and, as such, elaborate diagnostic and treatment algorithms are needed. Finding the adequate treatment intensity is fundamental to guarantee the best chance for cure as well as an appropriate long-term quality of life. De-intensification based on HPV evidence is evidently the main current focus of research, but it may not be the only approach; future studies will need to further expand the knowledge we tried to summarize in this brief review of literature, generating stronger prospective evidence and, hopefully, validating many of the proposed biomarkers, in order to allow for a better integration of the numerous clinical and biological aspects.
